# The Matron's Department

**Published:** 1905-10-07

**Authors:** 


### EDITOR'S LETTER-BOX.

[Our Correspondents are reminded that prolixity is a great bar to publication, and that brevity of style and conciseness of statement greatly facilitate early insertion.]

### "THE MATRON'S DEPARTMENT."

*To the Editor of* "The Hospital."

"A Matron" writes: I should like, through the medium of your correspondence columns, to express my appreciation of the most valuable articles appearing in the "Matron's Department," and to voice the thanks of, I am sure, many of my co-workers to the writer.

Books there are in profusion for the probationer, the nurse, the sister, but hitherto scant advice and little help has been accorded to those who, perhaps, need it more in their isolated positions than anyone. Instead, sharp and bitter criticism has often been the lot of the matron, and, compared with her, the scapegoat in the wilderness may be considered to have had a fairly easy time.

She is supposed to know by instinct exactly how to be a good housekeeper, an excellent organiser, a firm disciplinarian, an able teacher and lecturer. She must keep order, preserve unity, peace, and concord among her medical, nursing, and domestic staff; she must endear herself to every heart in the hospital; make both ends meet when a deficiency occurs either of stores or nurses; find "specials" where specials are not; be a filler-up of gaps; an even dispenser of justice and mercy; uniformly polite and even charming to the captious and complaining governor, doctor, nurse, subscriber, or justly irate "friend of the patient." She must see cherished ideals of carefully-thought-out improved nursing or domestic arrangements swept away by the decision of a single committee meeting, and meet it all with a pleasant smile and an entirely fresh set of plans. She has many difficulties that are all her own, and with which the excellent and lucid articles in the "Matron's Department" now help her successfully to grapple.

They show a clear insight into the exigencies of the case, and such a reasonable grasp of the whole position as cannot fail to be of the utmost practical use to all matrons reading them, and I greatly hope they will afterwards be published in book form for still wider circulation.

